# Galangin as a direct inhibitor of vWbp protects mice from *Staphylococcus aureus*‐induced pneumonia

**DOI:** 10.1111/jcmm.17129

**Published:** 2021-12-20

**Authors:** Yingli Jin, Panpan Yang, Li Wang, Zeyuan Gao, Jia Lv, Zheyu Cui, Tiedong Wang, Dacheng Wang, Lin Wang

**Affiliations:** ^1^ Key Laboratory of Zoonosis Research Ministry of Education/Institute of Zoonosis/College of Veterinary Medicine Jilin University Changchun China; ^2^ Department of Pharmacology College of Basic Medical Science Jilin University Changchun China; ^3^ College of Animal Science Jilin University Changchun China; ^4^ Changchun University of Chinese Medicine Changchun China; ^5^ Liaoning University of Traditional Chinese Medicine Dalian China

**Keywords:** galangin, pneumonia, *Staphylococcus aureus*, von Willebrand factor‐binding protein

## Abstract

The surge in multidrug resistance in *Staphylococcus aureus* (*S*. *aureus*) and the lag in antibiotic discovery necessitate the development of new anti‐infective strategies to reduce *S*. *aureus* infections. In *S*. *aureus*, von Willebrand factor‐binding protein (vWbp) is not only the main coagulase that triggers host prothrombin activation and formation of fibrin cables but also bridges the bacterial cell wall and von Willebrand factor, thereby allowing *S*. *aureus* to bind to platelets and endothelial cells, playing a vital role in pathogenesis of *S*. *aureus* infections. Here, we have identified that galangin, a bioactive compound found in honey and *Alpinia officinarum* Hance, is a potent and direct inhibitor of vWbp by coagulation activity inhibition assay, thermal shift assay and biolayer interferometry assay. Molecular dynamic simulations and verification experiments revealed that the Trp‐64 and Leu‐69 residues are necessary for the binding of galangin to vWbp. Significantly, galangin attenuated *S*. *aureus* virulence in a mouse *S*. *aureus*‐induced pneumonia model. In addition, we also identified that galangin can enhance the therapeutic effect of latamoxef on *S*. *aureus*‐induced pneumonia. Taken together, the results suggest that galangin may be used for the development of therapeutic drugs or utilized as adjuvants to combine with antibiotics to combat *S*. *aureus*‐related infections.

## 1 INTRODUCTION

*Staphylococcus aureus* (*S*. *aureus*) is a common opportunistic human pathogen that colonizes one‐third of the human population worldwide and causes various infections ranging from mild soft tissue infections to severe invasive bacterial infections such as endocarditis, necrotic pneumonia and osteomyelitis.^1^, ^2^, ^3^ The emergence and spread of multidrug‐resistant strains such as methicillin‐resistant *S*. *aureus* (MRSA) poses an increasing threat to public health.^4^, ^5^ Therefore, there is an urgent need to develop alternative antibacterial strategies to resist the increasingly serious multidrug‐resistant *S*. *aureus* infections.

*Staphylococcus aureus* causes disease via a large group of virulence factors, including surface‐associated adhesins and secreted proteinaceous toxins.^6^ Numerous experiments have confirmed that interfering with *S*. *aureus* virulence is a compelling approach for combating *S*. *aureus*‐associated infections because it exerts less evolutionary pressure on bacteria than traditional strategies, thereby reducing the risk of development of resistance.^7^, ^8^

A distinctive feature of coagulase‐positive *S*. *aureus* isolates is their ability to clot blood. This phenotype is caused by coagulase (Coa) or von Willebrand factor‐binding protein (vWbp), both of which can bypass the physiological coagulation cascade and directly activate thrombin.^9^ During host infection, either Coa or vWbp can bind to exosite I of prothrombin to form a coagulase‐prothrombin complex referred to as staphylothrombin, in which Coa or vWbp activates prothrombin by changing its conformation.^10^ Active staphylothrombin converts fibrinogen to insoluble fibrin,^11^ forming a fibrin meshwork. Staphylothrombin‐mediated fibrin generation contributes to colonization by *S*. *aureus* of the vascular wall, evasion of immune killing and spread of *S*. *aureus* via the bloodstream to all organ systems.^12^ The inhibition or deletion of coagulases can significantly reduce the disease‐causing potential of *S*. *aureus* and ameliorate disease progression in subcutaneous abscess sepsis, catheter infection and endocarditis in preclinical disease models,^13^, ^14^ indicating that *S*. *aureus* coagulases are appealing druggable targets for the treatment of *S*. *aureus* infections. Furthermore, the absence of Coa or vWbp does not affect the growth of *S*. *aureus*, and inhibition of these proteins does not exert selection pressure to promote the development of resistance. Therefore, inhibitors of coagulases have great research value for the treatment of *S*. *aureus* infections.^15^

**FIGURE 1:**
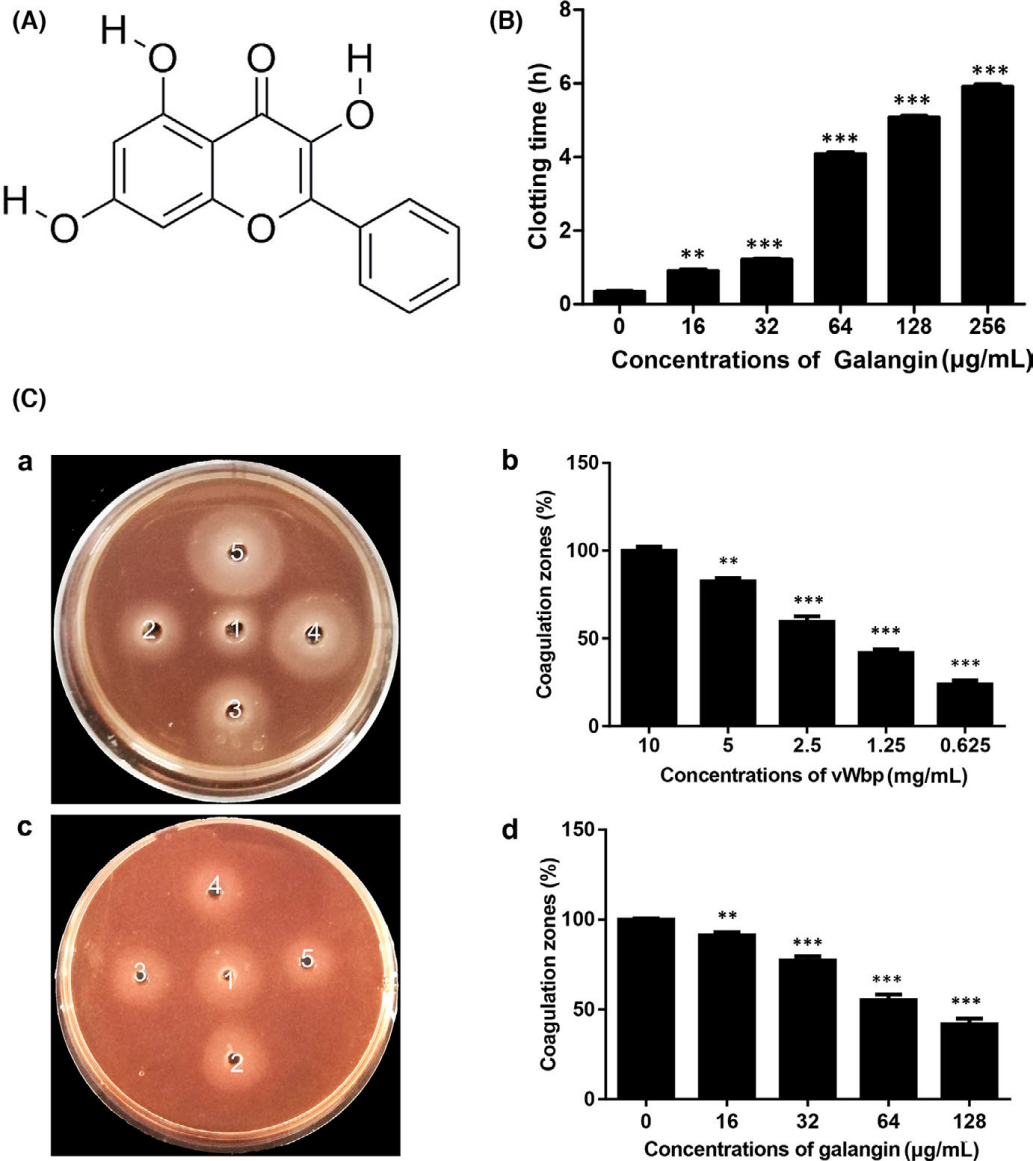
Galangin inhibits the activity of vWbp. (A) Structure of galangin. (B) Effects of various concentrations of galangin (0, 16, 32, 64, 128 and 256 μg/ml) on the coagulation activity of recombinant vWbp. (C) a, Clotting activity of different concentrations of vWbp (from well 5 to 1 are 10, 5, 2.5, 1.25 and 0.625 mg/mL, respectively.); c, 5 mg/ml vWbp was mixed with different concentrations of galangin (0, 16, 32, 64 and 128 μg/ml) and added to wells 1–5. Recording of the size of the coagulation zone (b, d). ^**^ *p* < 0.01 and ^***^ *p* < 0.001 were calculated using one‐way ANOVO

Although coagulase has been recognized as an important virulence factor of *S*. *aureus*, to date, few effective clinical inhibitors of coagulase have been reported. Our preliminary work of anticoagulation test identified galangin as an anti‐Sa‐vWbp molecule from 232 traditional Chinese medicines stored in our laboratory. In this study, we found that galangin (3,5,7‐trihydroxyflavone) (Figure 1A), a natural dietary flavonoid found in honey and *Alpinia officinarum* Hance, can inhibit the vWbp‐mediated clotting of blood by directly binding to vWbp. We systematically investigated the inhibitory mechanisms of galangin on vWbp and evaluated the therapeutic effect of galangin on *S*. *aureus*‐induced pneumonia.

## 2 MATERIALS AND METHODS

### 2.1 Bacterial strains, growth conditions and chemicals

The reference strains used in this study were *S*. *aureus* Newman D2C (ATCC 25904). The *vWbp* mutant strain (Δ*vWbp*) was constructed previously^16^ and stored in the laboratory. *Staphylococci* were cultivated in brain‐heart infusion (BHI) broth or on tryptone soya broth (TSB) agar plates at 37°C. *E*. *coli* strains DH5α and BL21 (DE3) were grown in Luria‐Bertani (LB) broth or on LB agar plates at 37°C with agitation. When necessary, ampicillin (100 μg/ml) was added to the LB broth, while chloramphenicol (10 μg/ml) was added to the BHI broth. Galangin was acquired from Chengdu Ruifensi Biotech Company.

### 2.2 Minimum inhibitory concentration and growth curve

The minimum inhibitory concentration (MIC) for galangin against *S*. *aureus* Newman was determined using the microdilution method in accordance with the standard M100‐S15 proposed by the Clinical and Laboratory Standards Institute (CLSI).^17^ For growth curve plotting, an overnight culture of *S*. *aureus* was inoculated into fresh BHI broth (1:100) containing 256 μg/ml galangin and incubated at 37°C for 24 h. The absorbance values were measured at 600 nm.

### 2.3 Cloning, expression and purification of recombinant vWbp

The DNA sequence encoding vWbp was amplified by polymerase chain reaction (PCR) from genomic DNA from the *S*. *aureus* Newman strain using the primer pair vWbp‐F/vWbp‐R. The PCR product was digested with BamHI and XhoI and cloned into the pET15b expression vector via the same enzyme restriction sites, yielding pET15b‐*vWbp*. After confirmation by DNA sequencing, pET15b‐*vWbp* was transformed into the *E*. *coli* BL21 (DE3) strain. Briefly, overnight cultures of BL21 (DE3) cells were diluted in LB broth to obtain an OD_600_ of ~0.1 and cultured until an OD_600_ between 0.6 and 0.8 was reached. The expression of recombinant vWbp was induced with 0.5 mM isopropylthio‐β‐D‐galactoside (IPTG) for an additional 12 h at 16°C. The cells were harvested, and the bacterial sediment was resuspended in a buffer containing 0.1 M Tris‐HCl (pH 7.5) and 0.5 M NaCl and lysed by sonication. The lysates were subjected to centrifugation at 12,000 *g* for 60 min. The recombinant His‐tagged vWbp protein was purified by a 6 × His/Ni‐NTA system (His Trap; GE Healthcare) as described previously.^18^ In addition, site‐directed mutagenesis to produce the substitutions D70A, W64A, L69A and M83A in vWbp was performed with a site‐directed mutagenesis kit (TransGen Biotech). All primers are shown in Table 1. The mutations were determined by DNA sequencing, and the detailed protein expression and purification process used for the mutant proteins was identical to that used for the wild‐type (WT) protein.

**TABLE 1:** Primers used in this study

Primer name	sequence(5′−3′)
vWbp‐F	GAACTCGAGGCATTATGTGTATCACAAATTTGGG
vWbp‐R	GAAGGATCCGCAGCCATGCATTAATTATTTGCC
W64A‐vWbp‐F	TCCAAACTTAGATAATGAAAGATTTGATAATC
W64A‐vWbp‐R	AAGGACGCTATTAAATCATCCAAGC
L69A‐vWbp‐F	CAAACGCAGATAATGAAAGATTTGATAATC
L69A‐vWbp‐R	GAAAGGACCATATTAAATCATCCAA
D70A‐vWbp‐F	TTAGCAAATGAAAGATTTGATAATC
D70A‐vWbp‐R	GTTTGGAAAGGACCATATTA
M83A‐vWbp‐F	AAGCTGCGAAAAAATATCAACA
M83A‐vWbp‐R	CTTTATATTCAGGATTATCAAATCTTTCAT

### 2.4 In vitro coagulase activity assays

To evaluate the coagulation activity of recombinant vWbp, plate and tube coagulase activity assays were conducted. For tube coagulase activity assays, 10 μl of purified vWbp in PBS (100 μM) was mixed with 190 μl of anticoagulated rabbit blood with heparin sodium, and 2.5 μl of different concentrations of galangin in glass tubes. The final concentrations of galangin were set as 0, 16, 32, 64, 128 and 256 μg/ml. Blood clotting was observed by tilting the tubes after incubation at 37°C and recording the time required for coagulation. For the plate coagulation assay, sterile agarose plates containing 0.9% agarose, 0.4% PEG 8000, 3 mg/ml bovine fibrinogen and 1% rabbit blood were prepared, and wells that were approximately 1 mm in diameter were punched into the agarose plates with a gel puncher before the assay. Different concentrations of recombinant protein were distributed into the wells in the agarose plates. The precipitation of fibrin was measured after incubation of the plates at 37°C overnight.

### 2.5 Preparation of a polyclonal antiserum against vWbp

Domestic rabbits were immunized with 0.4 mg of recombinant vWbp combined with Freund's complete adjuvant by multisite subcutaneous injection. Two boosters of 0.4 mg of vWbp with Freund's incomplete adjuvant were administered similarly at 2‐week intervals. After completing the immunization procedure, the rabbits were anaesthetized, and the blood sera were collected by carotid intubation and stored in 0.1% sodium azide at –20°C.

### 2.6 Western blot analysis

*Staphylococcus aureus* Newman was cultured with or without galangin until an OD of ~0.8 was reached. Bacterial cultures were collected, washed twice with PBS and homogenized at 4°C in lysis buffer with 10 mg/ml lysozyme and 40 mg/ml lysostaphin. Equal amounts of cell lysates (30 μg) were separated using a 10% (w/v) SDS‐PAGE gel, and the proteins were transferred to a polyvinylidene difluoride (PVDF) membrane. The membrane was then immersed in Tris‐buffer solution (TBS) containing 5% (wt/vol) nonfat milk and 0.1% Tween 20 overnight at room temperature. After blocking, the membrane was washed three times with TBST (TBS with 0.05% Tween 20) and incubated with the polyclonal antiserum against vWbp (1:500) for 2 h. After washing, the blot was hybridized with horseradish peroxidase (HRP)‐conjugated secondary goat anti‐rabbit antibody, and the protein bands were detected by a gel imaging system. Images were captured using a KODAK DCS 315 digital camera.

### 2.7 Thermal shift assays

The thermal shift assay was performed with a Bio‐Rad iQ5 real‐time PCR instrument (Bio‐Rad). Appropriate volumes of vWbp and 100 × Sypro Orange (Invitrogen) were mixed together, and then, 6 μl of the mixture, 4 μl of galangin and 10 μl of buffer solution containing 150 mM NaCl and 10 mM HEPES [pH 7.5] were mixed and distributed into qPCR tubes. Each tube contained 20 μl of solution containing 2 μg of vWbp and 1 μl of 100 × Sypro Orange, and the resultant molar ratio of vWbp to galangin was 1:10. After mixing, the tubes were covered with foil and incubated on ice for 10 min before fluorescence measurement. The tubes were heated from 25 to 95°C with a heating rate of 1°C/min. The fluorescence values were recorded every 10 s. Tubes containing the same constituents except galangin served as the reference control. All experiments were performed in triplicate.

### 2.8 Biolayer interferometry assay

The interaction of galangin and the vWbp protein was studied by an Octet RED biolayer interferometry (BLI) assay as previously described.^19^ Briefly, assay buffer (PBS containing 0.1% BSA and 0.02% Tween 20, pH 6.5), the vWbp protein (25 μg/ml) and different concentrations of galangin (7.81, 15.6, 33.3, 62.5 and 125 μM) were sequentially added to each well of a 96‐well opaque black plate. The Ni‐NTA biosensor was prewetted with assay buffer for 10 min before starting the assay and was then rinsed in the same buffer for 60 s to generate a baseline. The biosensor tip was immersed in the corresponding wells and incubated for 3600 s to allow protein binding. Unbound protein was removed by immersing the sensor in assay buffer for 300 s. Subsequently, the biosensor was exposed to different concentrations of galangin. The order of detection was baseline 60 s, association 200 s and dissociation 100 s. Biosensors coated with the protein without loading galangin were used as a control. Data collection was performed with Forte'Bio analysis software (v6.4) and analysed according to both references.

### 2.9 Molecular modelling of vWbp‐galangin interactions

The *S*. *aureus* vWbp homology model was built according to the homology of this vWbp with the three‐dimensional (3D) structure of the staphylocoagulase‐thrombin complex (PDB ID: 1NU7). The 3D structure of galangin was drawn with ChemBioDraw Ultra 14.0 (Cambridge Soft) and ChemBio3D Ultra 14.0 (PerkinElmer Inc.) software. The standard docking procedure for the protein and galangin was performed with the AutoDockTools 1.5.6 package^20^, ^21^ A molecular docking study was first employed to probe the binding mode of galangin with vWbp. After docking, the best docked pose was chosen for a 30‐ns molecular dynamic simulation. The detailed processes of molecular modelling, molecular dynamics simulation and binding free energy calculation were conducted as described previously.^22^, ^23^

### 2.10 Fluorescence quenching assay

The binding constant (*K_A_*) values for galangin with the various proteins were detected using fluorescence quenching assays as previously described.^24^ The excitation wavelength was set at 280 nm, and the fluorescence spectra were scanned in the wavelength interval of 280–400 nm with excitation and emission slits of 5 nm. The fluorescence intensity value at 292 nm was recorded, and the *K_A_* was calculated.

### 2.11 Mouse model of *S*. *aureus*‐induced pneumonia

Animal experiments were carried out according to the ethical standards and approved protocols of the Animal Welfare and Use Committee of Jilin University. Inbred C57BL/6J mice aged 7 weeks were purchased from Liaoning Changsheng Biotechnology Co., Ltd. The *S*. *aureus*‐induced pneumonia model was established as described previously.^25^ *S*. *aureus* bacteria were cultured overnight in BHI broth at 37°C with shaking. Culture aliquots were collected by centrifugation when the OD_600_ reached ~1.0. Then, the cell pellets were washed three times with PBS and resuspended in 750 μl of sterile PBS. Mice were randomly divided into groups, ten animals per group. After anaesthesia via ether inhalation, each mouse was inoculated with 30 μl of the bacterial suspension in the left nare and then held upright for 1 min. For the survival experiments, mice were challenged with 30 μL (4 × 10^9^ CFU) of *S*. *aureus* Newman and were subcutaneously administered 100 mg/kg galangin or PBS containing 2% DMSO (mock‐treated mice) immediately after infection and thereafter at 8‐h intervals. Survival and health status were examined at 12‐h intervals for 96 h. For the bacterial load and histopathological experiments, mice were challenged with 30 μl (2 × 10^9^ CFU) of *S*. *aureus*, and the subsequent treatment was the same as that given in the survival experiment. The surviving mice were sacrificed by cervical dislocation after anaesthesia at 48 h post‐infection. The lungs of each mouse were excised under aseptic conditions and then weighed and homogenized in PBS. The serially diluted homogenate was spread on BHI agar plates, and the CFUs were enumerated to determine the staphylococcal burden in the lung tissues. For the histopathological analysis, the lungs were dissected and fixed in 10% formalin. After fixation, the organs were embedded in paraffin, and sections (6 μM thick) were prepared and then stained with haematoxylin‐eosin for microscopic analyses. In addition, the therapeutic effect of galangin or galangin combined with latamoxef on *S*. *aureus* infection was further investigated using the *S*. *aureus*‐induced pneumonia model. Latamoxef (75 mg/kg) was administered via intramuscular injection immediately after *S*. *aureus* infection. The other experimental procedures and test indicators were the same as those used in the above experiments.

### 2.12 Statistical analysis

GraphPad Prism 5.0 was used for analysis of the experimental data. Experimental data were evaluated using a one‐way ANOVO in SPSS 22.0 statistical software. A value of *p* < 0.05 was considered statistically significant.

## 3 RESULTS

### 3.1 Galangin is an inhibitor of vWbp

To investigate the inhibitory effect of galangin on the clotting ability of vWbp, a tube coagulase activity assay was first used to detect the effect of galangin against vWbp, as shown in Figure 1B. The clotting time varied with the concentration of the compound, it increased with the increase of galangin concentration, indicating that galangin can inhibit the coagulase activity of vWbp. The plate coagulation assay further quantified the inhibitory effect of galangin on coagulase activity. In this test, fibrinogen was converted to insoluble fibrin, forming an obvious milky precipitate. As shown in Figure 1C(a, b), the sizes of the fibrin precipitation areas increased with increasing vWbp concentration (ranging from 0.625–10 mg/ml). Based on this result, 5 mg/ml vWbp was used for further analysis. Serial dilutions of galangin (at final concentrations of 16, 32, 64 and 128 μg/ml) were mixed with 5 mg/ml vWbp. The mixtures were added to wells 2–5, and well 1 contained 2% DMSO as a negative control (Figure 1C‐c). After incubation, the size of the coagulation zones was measured (Figure 1C‐d), and the diameter of the coagulative zones of wells 2–5 decreased as the concentration of galangin increased. Taken together, these results demonstrated that galangin can inhibit the coagulase activity of vWbp.

### 3.2 Galangin does not affect the growth of *S*.* aureus*

**FIGURE 2:**
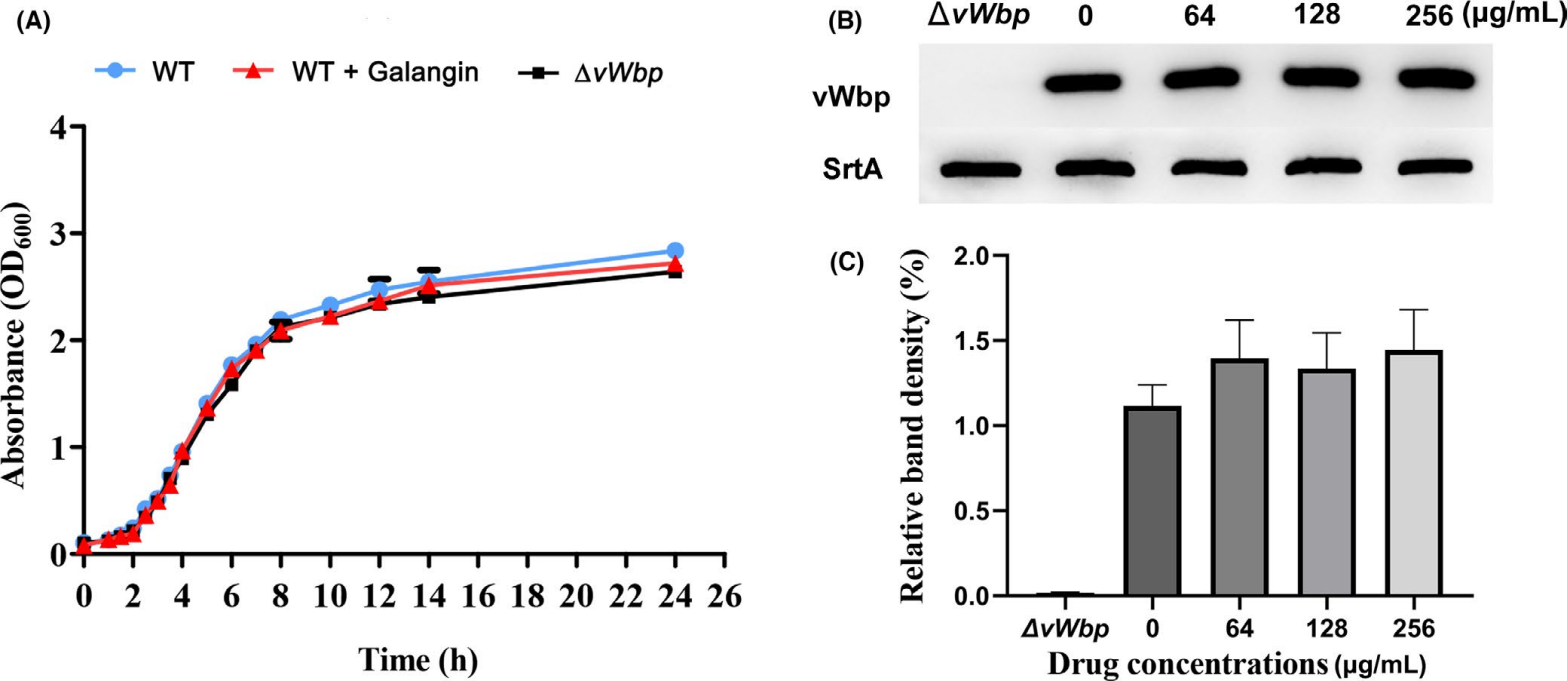
Galangin does not inhibit bacterial growth and the expression of vWbp. (A) Growth kinetics of *S*. *aureus* Newman treated with or without galangin (256 μg/ml). *S*. *aureus* Δv*Wbp* served as a control. Data represent in mean values ±SD. (B) Expression of vWbp was detected by Western blot. *S*. *aureus* Δ*vWbp* served as a control. Sortase A (SrtA) was used as the loading control in the experiment. The results are representative of two independent assays. (C) Grey value analysis of vWbp protein. Error bars indicate mean ±SD of three independent experiments

To test whether galangin influences the survival of *S*. *aureus*, the MIC of galangin against *S*. *aureus* was determined. The MIC value of galangin against the tested strains was greater than 512 μg/ml. Furthermore, the growth curve indicates that the growth status of *S*. *aureus* was not affected when 256 μg/ml galangin was present in the medium. No difference in the growth rate was observed between the *S*. *aureus* Δ*vWbp* strain and the WT strain (Figure 2A). Hence, galangin at a concentration lower than 256 μg/ml had no antibacterial effect on *S*. *aureus* in vitro.

### 3.3 Galangin does not interfere with the expression of vWbp

Western blot was performed to determine whether galangin influences the expression of vWbp. The total proteins from *S*. *aureus* cultured in the presence or absence of different concentrations of galangin (64, 128 and 256 μg/ml) were extracted, and the vWbp expression levels were checked with anti‐vWbp serum. As shown in Figure 2B, the vWbp expression levels of *S*. *aureus* treated with galangin were similar to those of untreated samples. Further measurement of the greyscale values of the vWbp bands showed that the expression levels of the protein did not change significantly (Figure 2C). The results showed that galangin did not affect the expression of vWbp.

### 3.4 Determination of the binding of galangin to vWbp

**FIGURE 3:**
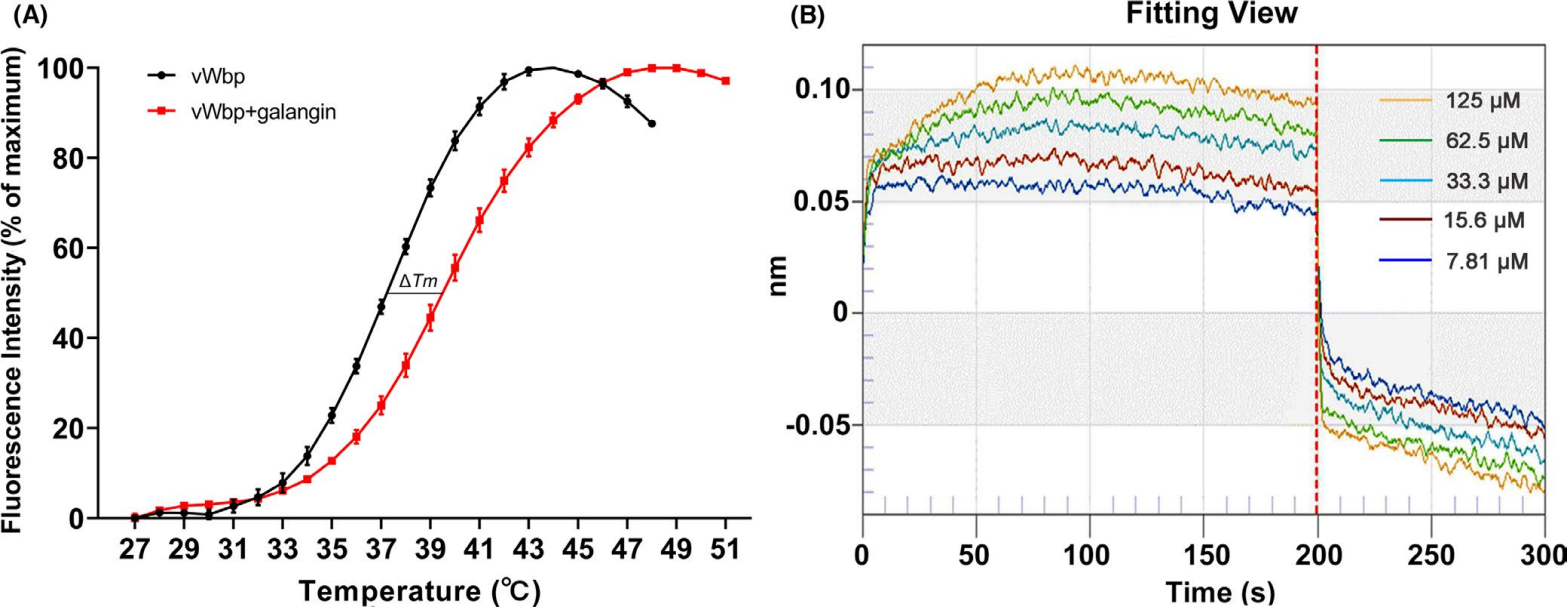
Determination of the binding of galangin to vWbp. (A) Galangin enhanced the thermal stability of the vWbp protein. The thermal shift assay of vWbp was performed in the presence (red curve) or absence (black curve) of galangin. (B) Biolayer interferometry assay showed that the association and dissociation sensorgrams for the interaction of different concentrations of galangin to vWbp. The Ni‐NTA biosensor was exposed to five different concentrations of galangin, and the fitting of the data to the curve was performed with Forte'Bio analysis software

The fluorescence‐based thermal shift assay (TSA) is a general method used for investigating protein‐ligand interactions based on the principle that the binding of small molecular compounds influences the thermal stability of proteins. Ligand binding usually induces a change in the conformational stability of proteins, which is reflected by a shift in the melting temperature (*T_m_*). A shift in the *T_m_* greater than 2°C indicates significant binding.^26^ To confirm the direct interaction between galangin and vWbp, the thermal stability of vWbp in the absence and presence of galangin was determined using TSA. As shown in Figure 3A, the addition of galangin shifted the *T_m_* of vWbp by 2.5°C, indicating that galangin directly binds to vWbp.

To further investigate the kinetic aspects of the interaction between galangin and vWbp, a BLI assay was performed. BLI is a reliable optical technique that investigates the interactions of proteins with other proteins or small molecules by analysing the interference pattern of the light reflected off the protein binding surface.^19^ In our study, we used a Ni‐NTA sensor to monitor the change in the layer thickness after the binding of small molecules to determine the binding strength. The sensorgrams of the association and dissociation of different concentrations of galangin with vWbp are shown in Figure 3B. Galangin showed slow association and dissociation patterns when binding to vWbp. The binding constant for the binding of galangin to vWbp was 11.0 μM, indicating that galangin binds strongly with vWbp.

### 3.5 Binding mode of galangin with vWbp

**FIGURE 4:**
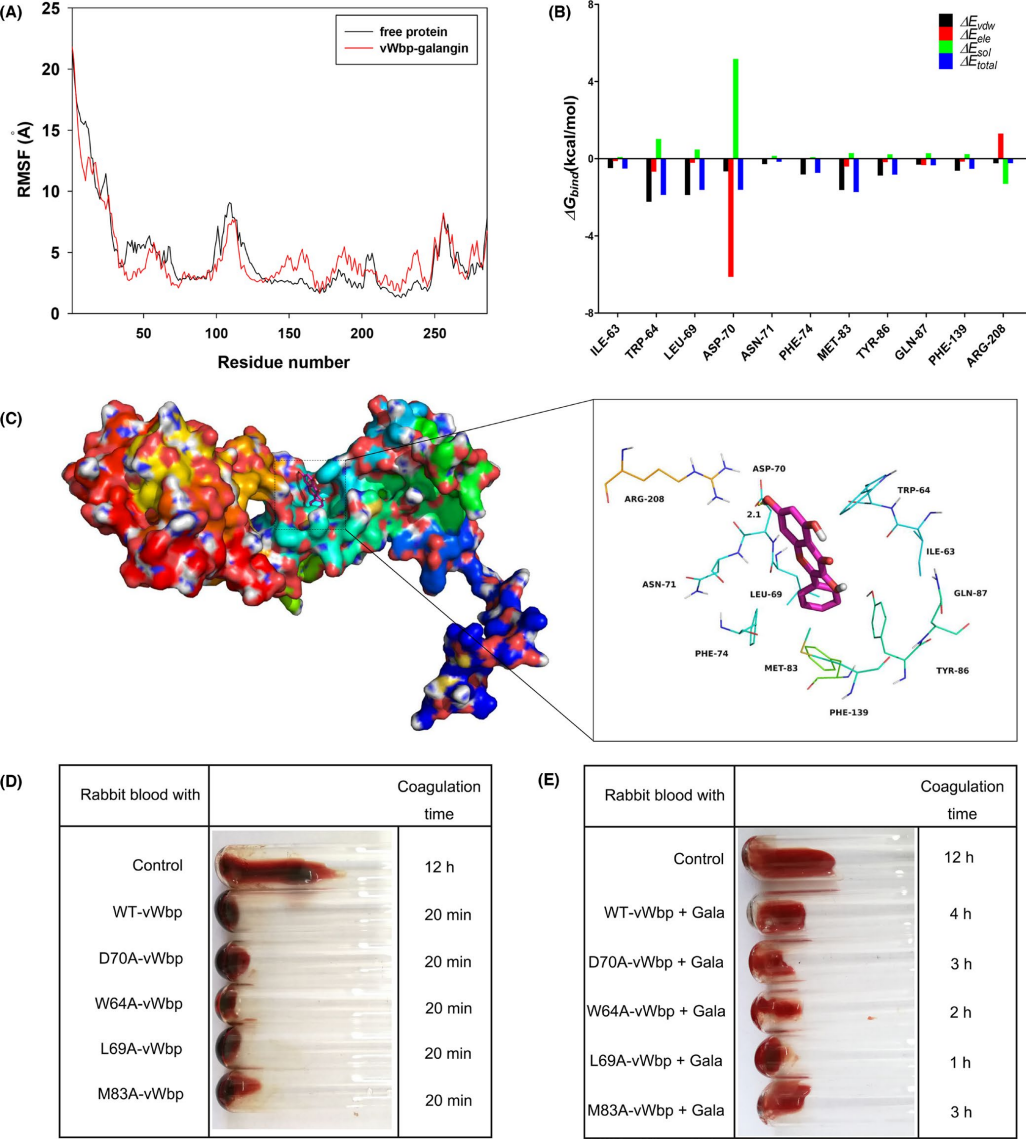
Binding mode of galangin with vWbp and verification of key residues involved in the interaction of inhibitor with vWbp. (A) RMSF of the residues of vWbp in the free protein and the vWbp‐galangin complex. (B) The binding energy decomposition of residues in the vWbp‐galangin complex. (C) The putative binding pattern of galangin and vWbp was obtained from an MD simulation. The clotting activity of WT vWbp and its mutant proteins in the absence (D) or presence (E) of galangin

Using a molecular modelling approach, we studied the binding mode of galangin with vWbp. The flexibility of the residues of the vWbp‐galangin system and those of free vWbp was studied according to the root mean square fluctuation (RMSF). As shown in Figure 4A, most of the residues in the vWbp binding sites showed a low degree of flexibility compared with that of free vWbp, with RMSF values of less than 5 Å, indicating that these residues are likely to become rigid due galangin binding. To study the energy contributions of residues within the binding site of the complex system, the contribution of each residue was calculated with the MMGBSA method. The calculation of the binding free energy indicated that electrostatic (∆*Eele*) and van der Waals (∆*Evdw*) forces are the main contributors to the binding of galangin and vWbp in the complex system (Figure 4B). The Asp‐70 residue makes a strong electrostatic (∆*Eele*) contribution, with a value of <–6.0 kcal/M. Further prediction of the binding patterns indicated that the Asp‐70 residue is close to the chromone group of galangin, leading to hydrogen bond formation (bond length: 2.1 Å) between vWbp and galangin (Figure 4C). Moreover, the Trp‐64, Leu‐69 and Met‐83 residues also contribute to the binding free energy, as these residues are close to galangin and have a significant van der Waals interaction with galangin. Subsequently, the total binding free energy of the vWbp‐galangin complex was calculated, and the estimated ∆*G_bind_* was –16.6 kcal/M, indicating that galangin can bind firmly to the binding site of vWbp.

### 3.6 Identification of binding sites in the vWbp‐galangin complex

Based on the predictions from molecular modelling, four vWbp mutants, namely, D70A‐vWbp, W64A‐vWbp, L69A‐vWbp and M83A‐vWbp, were constructed and expressed. The coagulation experiment showed that all four mutants displayed coagulation activity similar to that of WT vWbp, indicating that the amino acid mutations did not affect the clotting activity of vWbp (Figure 4D). However, the inhibitory effect of galangin on all mutants was significantly lower than its effect on WT vWbp. In particular, the sensitivity of W64A‐vWbp and L69A‐vWbp to galangin inhibition decreased most obviously (Figure 4E).

We studied the binding affinity of galangin to WT vWbp and its mutants by a fluorescence quenching assay. The binding constant (*K_A_*) between galangin and WT vWbp and the mutants was calculated. The results showed that the values of *K_A_* between galangin and the vWbp mutants all decreased, and the *K_A_* for W64A‐vWbp and L69A‐vWbp decreased most significantly (Table 2). We further performed the thermal shift assay for the four mutant proteins with or without galangin (Table 3). The addition of galangin increased the *Tm* of mutant proteins but at a lower level as compared with WT. L69A‐VWbp (±) galangin and W64A (±) galangin showed less *Tm* shift than that of D70A‐VWbp (±) galangin and M83A (±) galangin (Table 3), which was consistent with the results of the fluorescence quenching assay. These results indicated that L69 and W64 were the potential key sites for the binding of galangin to vWbp.

**TABLE 2:** Values of the binding constants (*K_A_*) based on the fluorescence quenching

Proteins	WT vWbp	D70A	W64A	L69A	M83A
*K_A_* (1 × 10^4^) L/mol	14.5 ± 1.34	10.3 ± 1.27	5.0 ± 1.59	3.2 ± 1.66	13.6 ± 1.03

**TABLE 3:** Thermal stability of vWbp mutant proteins with or without galangin

Proteins	vWbp^a^	L69A	W64A	D70A	M83A
Tm	37.0℃	35.0℃	35.4℃	35.5℃	35.7℃
Tm (+ galangin)	40.0℃	36.1℃	36.4℃	37.3℃	37.8℃
△Tm	3.0℃	1.1℃	1.0℃	1.8℃	2.1℃

^a^ The Tm values of vWbp are from Figure 3A.

### 3.7 Galangin has a protective effect against *S*. *aureus*‐induced pneumonia

**FIGURE 5:**
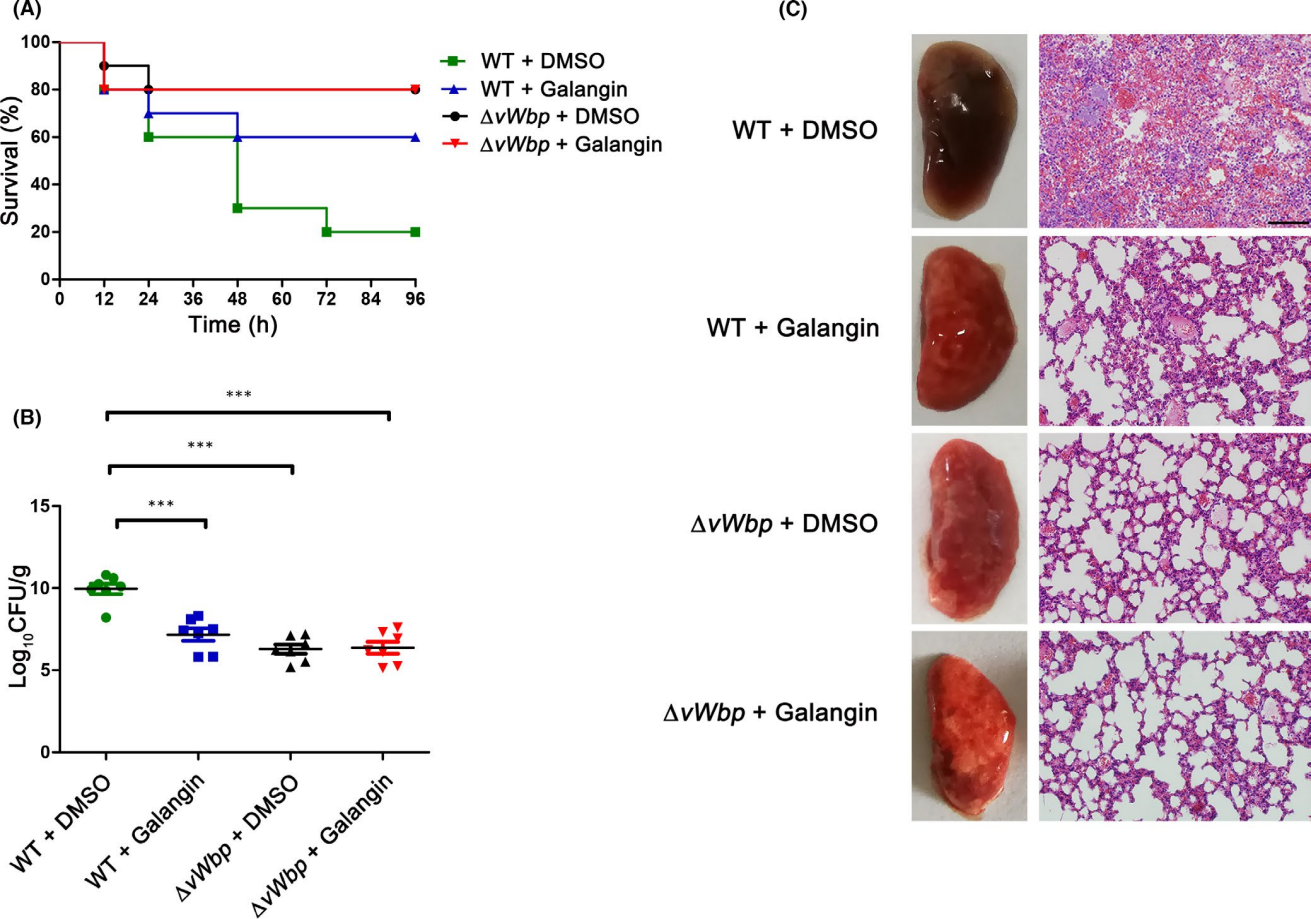
Galangin has a protective effect on *S*. *aureus* infection in mice. (A) Mice were infected with *S*. *aureus* Newman or *S*. *aureus* Newman Δ*vWbp* via the intra‐nasal route. Survival rates of galangin‐treated or untreated mice. WT + DMSO versus WT + galangin, **p < 0.01, log‐rank test. (B) The numbers of bacteria in the lungs of infected mice 24 h of infection. ^***^p < 0.001 versus DMSO‐treated mice (n = 10). *p* Values were calculated using one‐way ANOVO. (C) Gross pathological changes and histopathology of lung tissues in mice in different infected or treated groups. Scale bar, 100 μm. Animal data were obtained from two independent experiments

Given that coagulase‐positive *S*. *aureus* strains are highly pathogenic and can cause fatal suppurative pneumonia^27^ and that vWbp is an important virulence determinant in *S*. *aureus* infections,^28^ we speculated that inhibition of vWbp may prevent mice from developing *S*. *aureus*‐induced pneumonia. The therapeutic effects of galangin on *S*. *aureus* infection in vivo were evaluated using a mouse model of *S*. *aureus*‐induced pneumonia. In agreement with our speculation, treatment with galangin increased the survival rate of the group infected with *S*. *aureus* in comparison with the DMSO‐treated group (Figure 5A), and the difference was significant (60% vs. 20%, n = 10 per group). In addition, treatment with galangin led to a significant decrease (*p* < 0.001) in viable *S*. *aureus* bacteria in the lungs of mice treated with galangin compared with that in the control group (Figure 5B). To assess the pathological relevance of galangin protection, histopathological analysis of the lung specimens was performed. As shown in Figure 5C, the lungs of infected mice were red and hard; however, the lungs of uninfected mice and galangin‐treated mice were pink and spongy. Histopathological examination revealed severe alveolar destruction and large numbers of inflammatory cells in the lung tissues of DMSO‐treated infected animals, while mice treated with galangin showed a reduction in inflammation of the lungs, which manifested as reduced accumulation of inflammatory cells. In addition, there were no significant differences between the Δ*vWbp* group and the galangin treatment group. Together, these data established that galangin was a potent therapeutic agent against *S*. *aureus*‐induced pneumonia.

### 3.8 The combination of galangin with latamoxef has an improved therapeutic effect on *S*. *aureus*‐induced pneumonia

**FIGURE 6:**
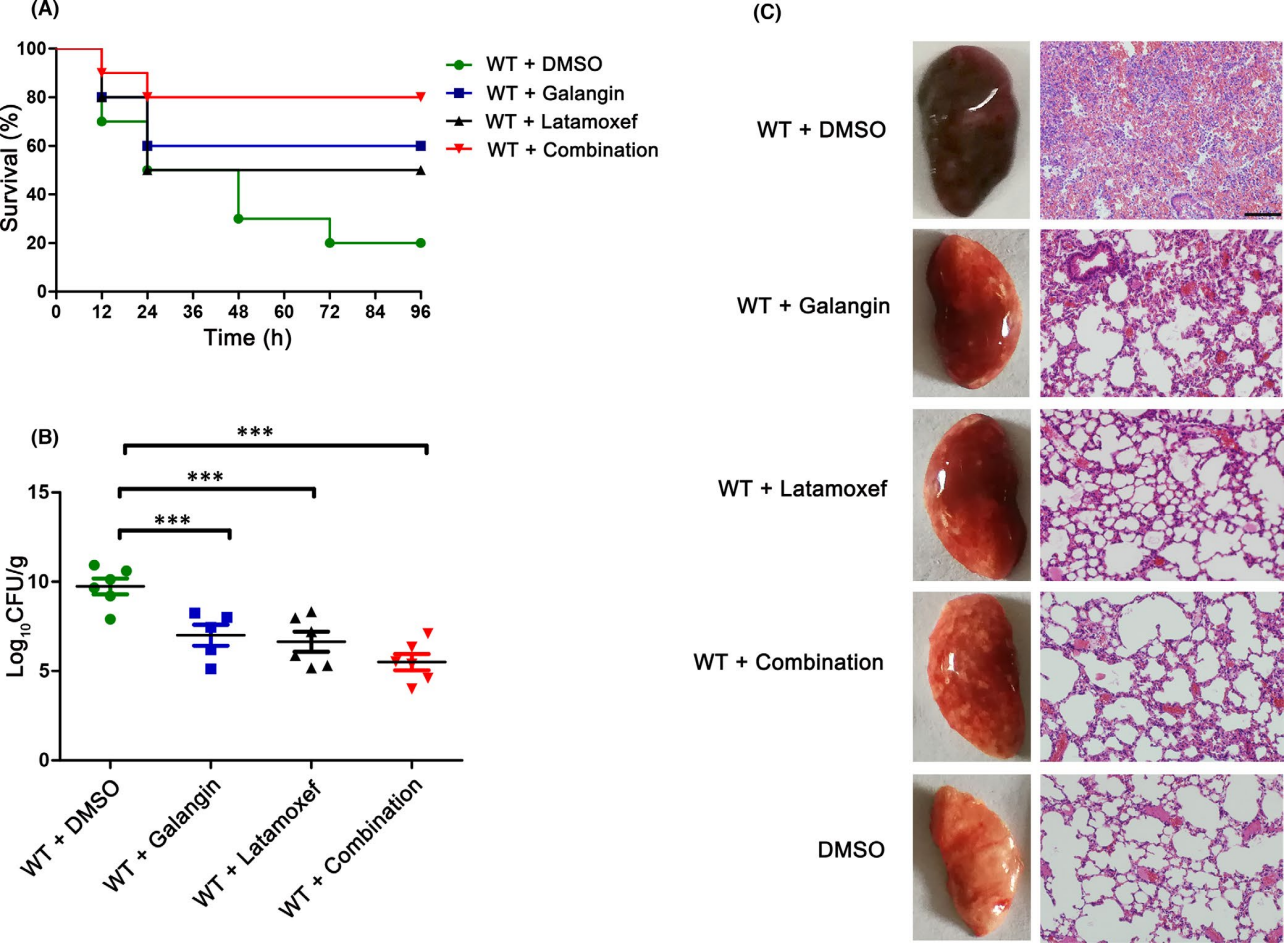
Galangin enhances the therapeutic effect of latamoxef on *S*. *aureus* infection. (A) Survival curves of mice that treated with or without galangin and/or latamoxef. WT + DMSO versus WT + combination, **p < 0.01; WT + galangin versus WT + combination, *p < 0.05; WT + latamoxef versus WT + combination, *p < 0.05, log‐rank test. (B) Bacteria burden in lungs of infected mice 24 h post‐infection. ^***^ *p *< 0.001 versus the DMSO group (n = 10). (C) Gross pathological changes and histopathology of lung tissues treated with either galangin and/or latamoxef. Scale bar, 100 μm. Animal data were obtained from two independent experiments

As an antivirulence agent, galangin disarms bacteria to reduce pathogenesis and promotes the elimination of bacteria by the host's immune system. However, for some immunocompromised patients, it is still difficult to eliminate the infection. Therefore, we further investigated the therapeutic effect of galangin combined with antibiotics on *S*. *aureus*‐induced pneumonia. Latamoxef is a semisynthetic oxacephalosporin antibiotic with similar antibacterial properties to third‐generation cephalosporins. It is used clinically to treat pneumonia, bronchitis and pleurisy caused by sensitive bacteria. Survival assays showed that 80% of the mice in the combination group survived, while only 20% of the mice in the untreated group survived; 60% of the mice survived in the galangin treatment group, and 50% of the mice in the latamoxef group survived (Figure 6A). The CFU count (Figure 6B) in the lungs of infected mice in the galangin treatment group (7.01 ± 0.59 log_10_ CFU/g) and the latamoxef treatment group (6.64 ± 0.56 log_10_ CFU/g) was observably lower than that in DMSO‐treated mice (9.74 ± 0.45 log_10_ CFU/g, *p* < 0.001). The combination treatment greatly reduced the number of bacteria in the lungs, and the CFU count in this group was the lowest (5.50 ± 0.46 log_10_ CFU/g). As shown in Figure 6C, the combination therapy significantly reduced lung tissue damage and inflammation compared with monotherapy. Together, these data suggest that galangin can enhance the efficacy of latamoxef and that the combination therapy had a better treatment effect on *S*. *aureus* infection than monotherapy.

## 4 DISCUSSION

In this study, we attempted to screen novel vWbp inhibitors from a library of phytochemicals from Chinese herbal extracts. We identified galangin as a potent inhibitor of vWbp in *S*. *aureus*. Galangin is a natural polyphenolic compound that possesses a variety of pharmacological activities, such as anti‐inflammatory,^29^ antioxidant^21^ and antifibrotic properties.^30^ Although galangin inhibited the clotting activity of vWbp, the addition of galangin to bacterial cultures (up to 256 μg/ml) did not affect the growth of *S*. *aureus* (Figure 2A) or the expression of vWbp (Figure 2B and 2C), indicating that galangin selectively inhibited the coagulase activity of vWbp and did not act by inhibiting the growth of *S*. *aureus* or vWbp expression. Moreover, the results of the TSA and the BLI assay demonstrated that galangin directly binds to vWbp (Figure 3). Molecular modelling revealed that the Asp‐70, Trp‐64, Leu‐69 and Met‐83 may play a key role in the binding of galangin to vWbp. Site‐specific mutagenesis analysis showed that mutation of Asp‐70, Trp‐64, Leu‐69 and Met‐83 did not affect the clotting activity of vWbp (Figure 4D), indicating that these amino acids that are adjacent to the binding pocket are not conformationally important for the activation of prothrombin by vWbp. Further coagulation activity inhibition assays showed that the inhibitory effect of galangin on the W64A‐vWbp and L69A‐vWbp mutants was significantly reduced compared with that on WT vWbp. Consistent with this result, the fluorescence quenching assay showed that the binding constant *(K_A_*) values of galangin for W64A‐vWbp and L69A‐vWbp were sharply decreased, indicating that mutation of the Trp‐64 or Leu‐69 residue reduced the affinity of galangin for vWbp. Based on these results, we confirmed that the amino acid residues Trp‐64 and Leu‐69 are the most important binding sites for the binding of galangin with vWbp.

*Staphylococcus aureus* is a common pathogen causing pneumonia. Among the major isolates obtained from pneumonia patients, MRSA accounts for approximately half of the cases initially diagnosed as pneumonia,^31^ leading to a reported mortality as high as 56%.^32^ It has been reported that the upregulation of key virulence factors may lead to increased toxicity and pathogenicity based on a model of pneumonia caused by MRSA.^33^ Sawai et al. found that coagulase may have contributed to infection with blood‐borne staphylococcal pneumonia.^28^ Consistent with their results, our previous findings established that vWbp plays a crucial role in *S*. *aureus*‐induced pneumonia, and knocking out the vWbp gene can dramatically improve the survival rate of mice infected with the Newman strain.^18^ The in vivo therapeutic effect of galangin on *S*. *aureus* infection was further evaluated using an *S*. *aureus*‐induced pneumonia mouse model. Galangin treatment increased the survival rate of mice challenged with lethal doses of *S*. *aureus* and reduced the lung bacterial load and inflammatory response compared with those in untreated mice (Figure 5), indicating that galangin treatment may significantly reduce the pathogenicity of *S*. *aureus*.

*S*. *aureus* infections often show reduced sensitivity to antibiotic treatment.^34^ Previous studies have shown that the combination of staphylothrombin inhibitors with antibiotics results in enhanced reduction in bacterial load in catheters and kidneys,^35^ indicating that antivirulence adjuvants could be used to increase the therapeutic efficacy of antibiotics. In this study, we found that galangin significantly improved the therapeutic effect of latamoxef. Compared with the monotherapy group, treatment with galangin combined with latamoxef significantly reduced the bacterial load in the lungs, the pathological changes in the lungs and the survival rate were significantly improved (Figure 6), suggesting that inhibition of vWbp by galangin can improve the efficacy of latamoxef against *S*. *aureus*‐induced pneumonia.

In conclusion, our findings demonstrated that galangin is a novel inhibitor of *S*. *aureus* vWbp and can be used alone or in combination with antibiotics to combat *S*. *aureus* infections.

## CONFLICT OF INTEREST

The authors confirm that there are no conflicts of interest.

## AUTHOR CONTRIBUTIONS

**Lin Wang:** Conceptualization (lead); Project administration (lead); Writing – review & editing (lead). **Dacheng Wang:** Funding acquisition (lead); Writing – review & editing (equal). **Yingli Jin:** Conceptualization (equal); Writing – original draft (lead). **Panpan Yang:** Formal analysis (equal); Investigation (lead); Writing – original draft (equal). **Li Wang:** Formal analysis (equal); Investigation (equal); Software (equal). **Zeyuan Gao:** Investigation (equal). **Jia Lv:** Investigation (equal). **Zheyu Cui:** Investigation (equal). **Tiedong Wang:** Writing – review & editing (equal).
